# Low‐Temperature Combustion Synthesis of a Spinel NiCo_2_O_4_ Hole Transport Layer for Perovskite Photovoltaics

**DOI:** 10.1002/advs.201701029

**Published:** 2018-03-03

**Authors:** Ioannis T. Papadas, Apostolos Ioakeimidis, Gerasimos S. Armatas, Stelios A. Choulis

**Affiliations:** ^1^ Molecular Electronics and Photonics Research Unit Department of Mechanical Engineering and Materials Science and Engineering Cyprus University of Technology Limassol 3041 Cyprus; ^2^ Department of Materials Science and Technology University of Crete Heraklion 71003 Greece

**Keywords:** combustion synthesis, hole transporting layers, NiCo_2_O_4_, perovskites solar cells, transparent conductive oxides

## Abstract

The synthesis and characterization of low‐temperature solution‐processable monodispersed nickel cobaltite (NiCo_2_O_4_) nanoparticles (NPs) via a combustion synthesis is reported using tartaric acid as fuel and the performance as a hole transport layer (HTL) for perovskite solar cells (PVSCs) is demonstrated. NiCo_2_O_4_ is a p‐type semiconductor consisting of environmentally friendly, abundant elements and higher conductivity compared to NiO. It is shown that the combustion synthesis of spinel NiCo_2_O_4_ using tartaric acid as fuel can be used to control the NPs size and provide smooth, compact, and homogeneous functional HTLs processed by blade coating. Study of PVSCs with different NiCo_2_O_4_ thickness as HTL reveals a difference on hole extraction efficiency, and for 15 nm, optimized thickness enhanced hole carrier collection is achieved. As a result, p‐i‐n structure of PVSCs with 15 nm NiCo_2_O_4_ HTLs shows reliable performance and power conversion efficiency values in the range of 15.5% with negligible hysteresis.

## 1 Introduction

Over the last few years, a great deal of effort has been made to improve photovoltaic performance based on organic–inorganic lead halide perovskites, which has been reported to exhibit power conversion efficiencies (PCEs) over 20%.^1^, ^2^, ^3^, ^4^, ^5^ The use of organic–inorganic lead halide perovskites has attracted intense interest due to extraordinary characteristics such as high light absorption,^6^, ^7^, ^8^, ^9^ enhanced charge transport properties, and direct band gap transition. For the fabrication of efficient perovskite solar cells (PVSCs), the so‐called n‐i‐p architecture is widely used.^10^ For the p‐i‐n‐type PVSCs, also called inverted architecture structure, poly(3,4‐ethylenedioxythiophene): poly(styrenesulfonate) (PEDOT:PSS) is commonly used as a hole transport layer (HTL). PEDOT:PSS is usually used as HTL for printed electronic due to its facile processing and good electrical conductivity and transparency.^11^, ^12^, ^13^, ^14^, ^15^, ^16^ On the other hand, the hydroscopicity and inhomogeneous electrical properties might limit its performance as a HTL for advanced optoelectronic applications.^17^, ^18^ Recently, p‐type metal oxides and complexes, such as NiO, V_2_O_5_, CuO, CuSCN, CuPc, and ZnPc,^19^, ^20^, ^21^, ^22^, ^23^, ^24^, ^25^ have been incorporated as HTLs into PVSCs. Inorganic p‐type semiconductor materials have the advantages of providing energy levels for improved hole selectivity and chemical stability, showing promising performance as HTLs in PVSCs.^26^, ^27^

Up to now, sol–gel method is the most commonly used technique for the fabrication of the p‐type metal oxides. However, in order to achieve the required crystallinity, temperatures above 400 °C are usually required. The need for high temperature is increasing the fabrication cost and limits their potential use for printed electronic applications.^28^ Thus, there is a demand for the development of metal oxides using preparation methods that require lower temperatures.

Among many processes for the synthesis of nanomaterial compounds, combustion synthesis emerges as an efficient alternative approach. The combustion synthesis, in principles, can be defined as a redox (reduction/oxidation) or electron transfer process, in which the fuel is oxidized (increase of the oxidation state) and the oxidizer is reduced (reduce of the oxidation state) in an exothermic reaction.^29^, ^30^, ^31^, ^32^ Various types of combustion synthesis processes have been applied to obtain nanoparticles (NPs) and they can be categorized according to the educts (gaseous, liquid, or solid) and the process (e.g., combustion synthesis in the gas, solid, or liquid phase, volume combustion synthesis, self‐propagating high‐temperature synthesis, etc.).^33^, ^34^, ^35^

The combustion technique appears to be versatile and effective for the synthesis of high crystallinity solution‐processed metal oxides thin films using low temperature.^36^, ^37^, ^38^, ^39^ Since it is an exothermic process, with a high heat release rate, the need for high temperatures is avoided and the production of high purity and homogeneous NPs formation is simultaneously achieved.^40^, ^41^, ^42^ For the production of metal oxides, liquid phase combustion synthesis has proven to be the most suitable, where usually metal salts (for instance nitrates) serve as oxidizers dissolved in saturated aqueous or alcoholic solutions in combination with organic fuels (e.g., urea, glycine, citric acid, and others).^35^, ^43^, ^44^ Upon heating gelation occurs and then combustion process starts resulting in the synthesis of the corresponding metal oxide.^33^, ^45^ The combustion synthesis of metal oxides exhibits great advantages comparing to other NP synthesis methods; namely, simple experimental setup, reduced number of postprocessing steps, formation of NPs without agglomeration, high purity of materials, and precise control of particle's size and crystallinity by adjusting the processing parameters.^30^, ^32^, ^46^, ^47^, ^48^, ^49^, ^50^ In general, the reaction mechanism of the combustion is affected by many factors such as the type of fuel, fuel‐to‐oxidizer ratio, ignition temperature, and the H_2_O content of the precursor blend.^33^, ^34^, ^51^, ^52^

Nickel cobaltite (NiCo_2_O_4_) is a p‐type transparent conductive oxide semiconductor consisting of abundant and environmentally friendly elements (Co, Ni), with a relatively wide optical band gap (≈2.1–2.4 eV), deep‐lying valence band (VB of 5.3 eV) that matches well with the VB of CH_3_NH_3_PbI_3_ perovskite semiconductor and a much better conductivity than NiO and Co_3_O_4_ (at least two orders of magnitude higher).^53^ These characteristics render NiCo_2_O_4_ one of the most promising candidates for electronic applications. NiCo_2_O_4_ adopts a cubic spinel structure in which all the Ni ions occupy the octahedral sites and the Co ions are distributed between the tetrahedral and octahedral sites.^53^, ^54^, ^55^ It possesses high physical and chemical stability which is a necessity for high‐performance electronic devices. These attractive features make NiCo_2_O_4_ an appropriate candidate material for introduction as HTL in PVSCs to achieve high‐performance photovoltaic devices. NiCo_2_O_4_ derivatives have been used previously in many other applications such as anodic oxygen evolution,^56^ inorganic and organic electrosynthesis,^57^ development of supercapacitors,^53^, ^58^ or infrared transparent conducting electrodes, sensors, optical limiters, and switches,^59^, ^60^ but before this publication not for any type of solar cells.

Up to now various low‐temperature synthetic routes such as hydrothermal, co‐precipitation,^61^, ^62^ and thermal decomposition of the precursors, such as hydroxide nitrates^63^, ^64^ and hydrazine carboxylate hydrates,^65^ have been developed for the synthesis of NiCo_2_O_4_. Moreover, nanostructured aggregates of NiCo_2_O_4_ have been synthesized by employing heterometallic alkoxide precursor in the presence of a supramolecular liquid.^66^ However, the production of high purity and monodispersed NPs with the above‐mentioned synthesis approaches has not been completely achieved.

In this work, we present a one‐step synthesis of low‐temperature solution‐processable nickel cobaltite (NiCo_2_O_4_) via combustion chemistry proposing for the first‐time tartaric acid as a fuel and nitrate as an oxidizer agent. NiCo_2_O_4_ NPs with an average size of ≈4 nm and narrow particle‐size distribution were prepared using a cost‐effective, low‐temperature combustion synthesis method calcinated at 250 °C for 1 h. Those ultrafine NPs enable the formation of compact, very smooth, high electrically conductive, and relatively optically transparent NiCo_2_O_4_ films, which were utilized, for the first time, as HTLs in a solution‐processed p‐i‐n PVSC. The effect of NiCo_2_O_4_‐NPs HTL thickness on PVSC characteristics is also investigated. A comparative study of devices incorporating different thickness of NiCo_2_O_4_‐HTLs reveal a difference in hole extraction efficiency. The photoluminance spectroscopy measurements on perovskite films showed a reduced electron–hole pair recombination for the optimized 15 nm thick NiCo2O4‐HTL. Additional electroimpedance spectroscopy and Mott–Schottky (M‐S) measurements on PVSC confirm the better hole extraction inducing an enhancement in the PVSC characteristics and negligible PCE hysteresis. The corresponding PVSC exhibits a high fill factor (FF) ≈ 80% and a reliable performance with PCE of 15.5%. The results demonstrate the great potentials of applying the low‐temperature combustion synthesis for fabrication of highly reproducible and reliable metal oxide NPs which can be used for the formation of HTLs in variety of solution‐processed printed electronic devices.

## 2 Results and Discussion

Combustion synthesis has been applied recently for the low‐temperature fabrication of metal oxide thin films.^67^ In general, solution combustion synthesis has the advantage of rapidly producing homogeneous metal oxide materials with fine grain size, and most significantly at much lower temperature compared with the conventional solid‐state reaction processes and co‐precipitation methods. The structural and morphological characteristics of the resulting materials closely depend on the type and amount of chemicals (fuel, oxidizing agent) used in the synthesis.^33^, ^45^ Furthermore, the choice of the fuel regent for the combustion process has an essential role to avoid the formation of large clusters or/and large voids between the grains.^46^ A fuel, i.e., the substance capable of acting as electrons acceptor, can significantly affect the properties of the final product, such as grain size, surface area, morphology, crystal phase, and degree and nature of particle agglomeration.^52^, ^55^

In this work, tartaric acid is proposed as a fuel that is critically important to obtain uniform single‐crystalline phase NiCo_2_O_4_ NPs. The advantage of using tartaric acid is related to the formation of heterometallic polynuclear complexes^68^, ^69^ due to the presence of its carboxylate and hydroxyl groups in a proper orientation, where the binding metal ions (i.e., Ni^2+^ and Co^3+^) come close together.^70^ Ultimately, the formation of nickel cobaltite NPs is the consequence of decomposition of polynuclear complexes upon mild heating in the presence of concentrated HNO_3_.^71^

### 2.1 Synthesis and Characterization NiCo_2_O_4_ NPs

It is well documented in the literature that thin metal oxide films can be obtained at temperatures lower than bulk‐like powders via combustion synthesis because of the enhanced gas transport mechanisms and the easier out‐diffusion of volatile products.^72^ This means that thin films may decompose at low temperature and exhibit high sensitivity to any residual reactive gas present in the oven.^67^, ^73^

**Figure 1:**
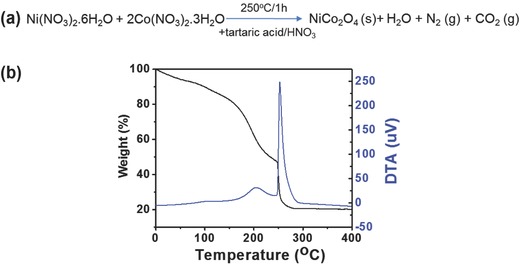
a) Depiction of the synthetic route for spinel NiCo_2_O_4_; b) TGA and DTA profiles of the as‐prepared NiCo_2_O_4_ via combustion process.

For the combustion synthesis of NiCo_2_O_4_, metal precursors (Ni and Co nitrates) and tartaric acid (at a 1:2 molar ratio, respectively) were dissolved in 2‐methoxyethanol containing a small amount of 0.24 × 10^−3^ m HNO_3_ (**Figure** **1**a). The combustion reaction of the Ni(II)/Co(III)‐tartaric complexes was monitored by differential scanning calorimetry (DSC) and thermogravimetric analysis (TGA), applying a heating rate of 10 °C min^−1^ in air. As shown in Figure 1b, the reaction exhibits an intense exothermic peak at ≈260 °C in the DSC curve, which coincides well with the abrupt mass loss (at ≈250 °C) observed in TGA curve. This implies that the formation of NiCo_2_O_4_ NPs via such combustion method can effectively proceed at a much lower temperature.

**Figure 2:**
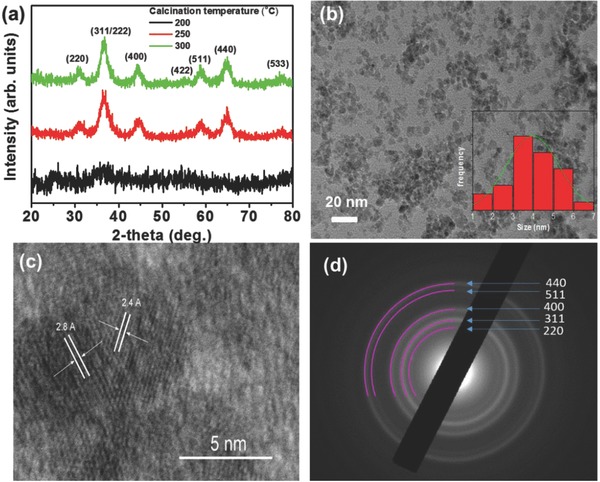
a) XRD patterns of NiCo_2_O_4_ NPs at 200 °C (black), 250 °C (red), 300 °C (green solid line) combustion temperatures. b) Representative TEM image (inset: particle size distribution plot of the NiCo_2_O_4_ NPs at 250 °C, showing an average diameter of 4 ± 1.3 nm), c) high‐resolution TEM, and d) SAED pattern of the as‐synthesized NiCo_2_O_4_ NPs obtained at 250 °C.

In order to crystallize the as‐synthesized films to spinel phase, 1 h of heating was applied at different temperatures, i.e., 200, 250, and 300 °C, and the X‐ray diffraction (XRD) results of the obtained materials are shown in **Figure** **2**a. The XRD patterns of the materials calcined at 250 and 300 °C correspond to the spinel phase of NiCo_2_O_4_, although with a larger grain composition for the sample treated at 300 °C, as indicated by the narrow full width of half‐maximum of XRD peaks. The angular position of the diffraction peaks matches well with standard XRD pattern of cubic spinel NiCo_2_O_4_ with JCPDS card no 20‐0781. Notably, we did not observe any additional peaks arising from impure phases, indicating the single‐crystalline nature of samples. The average grain of the NiCo_2_O_4_ NPs was estimated from the diffraction peak (220) by using the Scherrer's equation and was found to be ≈3.5 nm for the sample annealed at 250 °C and ≈5 nm for the sample annealed at 300 °C. In contrast, the XRD pattern of the material obtained after 200 °C heat treatment showed no diffraction peaks, indicating the formation of an amorphous structure.

Transmission electron microscope (TEM) verified the high crystallinity and phase purity of spinel NiCo_2_O_4_ NPs prepared by the low‐temperature combustion method. Figure 2b displays a typical TEM image of the NiCo_2_O_4_ NPs synthesized at 250 °C. It can be seen that this material is composed of individual NPs with an average diameter of 4 ± 1.3 nm, which is very close to the grain size calculated from XRD patterns. The high‐resolution TEM image shown in Figure 2c reveals that the NiCo_2_O_4_ NPs possess a single‐phase spinel structure with high crystallinity; combined with XRD results, the observed lattice fringes with interplanar distances ≈2.4 and ≈2.8 Å can be assigned to the (331) and (220) crystal planes of spinel NiCo_2_O_4_, respectively. The crystal structure of the NiCo_2_O_4_ NPs was further studied by selected‐area electron diffraction (SAED). The SAED pattern taken from a small area of the NiCo_2_O_4_ NP aggregates (Figure 2d) shows a series of Debye–Scherrer diffraction rings, which can be assigned to the spinel phase of NiCo_2_O_4_. No other crystal phases were observed by means of electron diffraction. In addition, characterization of the chemical composition of NiCo_2_O_4_ NPs with energy‐dispersive X‐ray spectroscopy (EDS) showed an overall Ni:Co atomic ratio close to 1:2, in agreement with the stoichiometry of NiCo_2_O_4_ compound (Figure S1, Supporting Information).

### 2.2 Blade Coating Processed Thin Films of NiCo_2_O_4_ NPs

**Figure 3:**
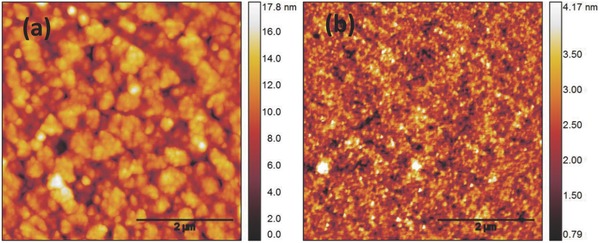
AFM images of a) ITO/NiCo_2_O_4_ and b) quartz/NiCo_2_O_4_ NPs thin films after combustion synthesis at 250 °C (the scale bar is 2 µm).

Thin films of NiCo_2_O_4_ NPs were produced on top of quartz and indium tin oxide (ITO) substrates using the doctor‐blading technique, the processing parameters are described within the Experimental Section.**Figure** **3** demonstrates the surface topography of a 15 nm thick NiCo_2_O_4_ film fabricated on top of glass/ITO and quartz substrates, as obtained by atomic force microscopy (AFM) scans. On top of ITO substrate (Figure 3a), the surface roughness is about 2.7 nm, while the film fabricated on quartz substrate (Figure 3b) exhibits an impressively smooth and compact topography of only 0.56 nm roughness. The development of a low roughness layer is a beneficial feature for the photovoltaic performance since it enables us to grow perovskite top layers with low roughness and enhanced homogeneity. Moreover, the dense NiCo_2_O_4_ NPs‐based thin film exhibits an increase electrical conductivity up to 4 S cm^−1^ at room temperature, measured using four‐point probe method.

**Figure 4:**
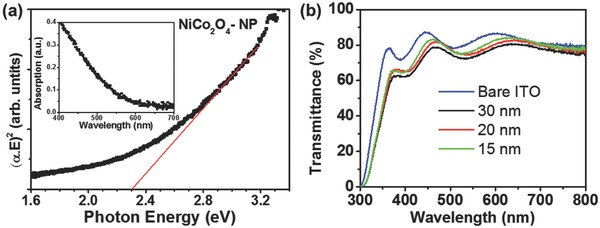
a) (*αE*)^2^ versus photon energy (eV) plot, showing an energy band gap of 2.32 eV. Inset: UV–vis absorption spectrum of NiCo_2_O_4_ NPs film fabricated on quartz substrate. b) Transmittance spectra of bare glass/ITO and NiCo_2_O_4_ NPs films deposited on glass/ITO substrate with thickness of 15, 20, and 30 nm.

**Figure** **4**a (inset) shows the optical absorption spectrum of the NiCo_2_O_4_ NPs film fabricated on a quartz substrate and the corresponding Tauc plot (Figure 4a) for direct allowed transition ((*αE*)^2^ vs. photon energy (*E*)), giving an optical band gap of 2.32 eV. NiCo_2_O_4_ thin films of different thicknesses were also fabricated on glass/ITO substrate in order to investigate the transmittance of the front contact at UV–vis spectrum. Figure 4b displays the transparency of bare glass/ITO and NiCo_2_O_4_ HTLs coated on glass/ITO substrate; it could be seen that NiCo_2_O_4_ films thinner than 20 nm reduce only slightly the transparency of the glass/ITO substrate for wavelengths longer than 450 nm, allowing more intense light to reach the absorbing layer.

### 2.3 Device Performance

**Figure 5:**
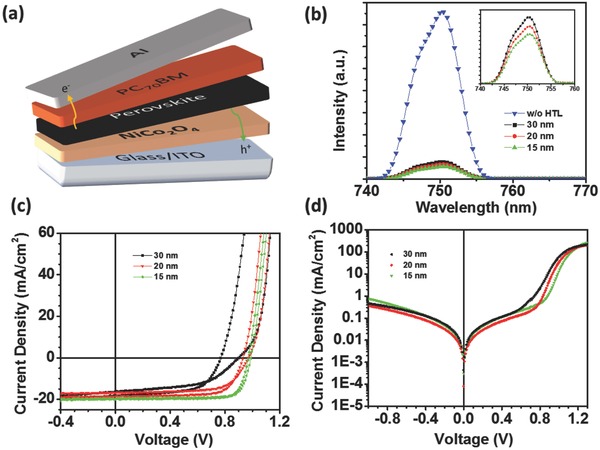
a) The structure of the p‐i‐n perovskite solar cells under study (ITO/NiCo_2_O_4_‐NPs/CH_3_NH_3_PbI_3_/PC[70]BM/Al). b) Photoluminescence (PL) spectra (inset: magnification of the PL spectra at lower intensities), and current density versus voltage (*J*–*V*) plots c) under 1 sun illumination and d) under dark conditions of the ITO/NiCo_2_O_4_‐NPs/CH_3_NH_3_PbI_3_ devices fabricated with NiCo_2_O_4_ with different thickness (15 nm, green solid line; 20 nm, red line; and 30 nm, black line).

Complete p‐i‐n architectures of PVSCs were fabricated employing NiCo_2_O_4_ HTLs with three different thicknesses, 30, 20, and 15 nm. On top of each NiCo_2_O_4_ HTL, a 230 nm thick perovskite film was solution processed as described in the Experimental Section. The deposited perovskite film exhibits a low roughness of 5.4 nm (Figure S2, Supporting Information) and a mean grain size of 0.22 µm with a standard deviation of 0.051 µm (Figure S3, Supporting Information), as calculated by AFM topography measurements. To complete the devices, a PC[70]BM film was spin‐coated on the top of the perovskite semiconductor serving as the electron transporting layer followed by a 100 nm thick thermally evaporated Al (**Figure** **5**a). Figure 5c depicts the device current density–voltage characteristic (*J*–*V* under calibrated AM1.5G illumination) of the PVSCs fabricated with 15, 20, and 30 nm thick NiCo_2_O_4_ HTLs, and the corresponding solar cell parameters are summarized in **Table** **1**, where the series resistance (*R*_s_) was extracted from the dark *J*–*V* curves (Figure 5d). It is observed that the *J*–*V* hysteric on the forward and reverse sweep is reduced as the thickness of NiCo_2_O_4_ decreases from 30 to 15 nm, while both the *V*_oc_ and FF increase. Concretely, for the reversed sweep the *V*_oc_ was increased from 0.90 to 0.99 V and the FF from 53.0% to 79.9%, while the hysteric on the PCE for the 15 nm thick NiCo_2_O_4_ layer is negligible. On the other hand, the short circuit current (*J*_sc_) showed the lowest increase (≈8%) for a forward sweep from 18.47 to 19.94 mA cm^−2^, compared to both *V*_oc_ and FF. Consequently, the device consisting of a 15 nm thick NiCo_2_O_4_ HTL exhibits a PCE as high as 15.5% for the forward sweep. The PCE of devices with thinner NiCo_2_O_4_ HTLs were declined (not shown here) exhibiting high leakage currents due to limitations of not fully covered ITO. Figure S4 (Supporting Information) demonstrates the external quantum efficiency (EQE) measurements of the corresponding devices. It is noticed that for 15 nm thick NiCo_2_O_4_ film the overall efficiency is increased comparing to thicker layers due to higher transmittance as well as to a better charge collection, as it will be shown below. All the devices show a declined performance at longer wavelength (600–750 nm) which can be attributed to the relatively thin perovskite layer (≈250 nm) and to not optimized electron carrier selectivity of the top electrode (PC[70]BM/Al) that used within this studies.^22^

**Table 1:** Extracted solar cell parameters from the *J*–*V* characterization of the ITO/NiCo_2_O_4_/CH_3_NH_3_PbI_3_/PC[70]BM/Al devices using NiCo_2_O_4_ NPs layers with different thickness

NiCo_2_O_4_	*J*_sc_ [mA cm^−2^]	*V*_oc_ [V]	FF [%]	PCE [%]	*R*_s_ [Ω cm^2^]
15 nm (forw.)	19.94	0.97	79.9	15.5	1.06
(rev.)	19.60	0.99	79.2	15.4	
20 nm (forw.)	18.47	0.93	73.2	12.6	1.34
(rev.)	16.83	0.97	67.8	11.1	
30 nm (forw.)	18.45	0.77	61.2	8.7	1.37
(rev.)	16.29	0.90	53.0	7.8	

The impact of the NiCo_2_O_4_ HTL thickness on the ITO/NiCo_2_O_4_‐NPs/perovskite device performance was evaluated by photoluminescence (PL) spectroscopy (see Figure 5b). Comparing to reference structure (without NiCo_2_O_4_ HTL), the PL signal of the devices with NiCo_2_O_4_ HTL show a quenching of more than 90%, indicating a great reduction in the band‐to‐band charge recombination and, thus, a better hole carrier selectivity of the ITO electrode covered by NiCo_2_O_4_. Further, the PL intensity is lower in 15 nm NiCo_2_O_4_ film than that of the thicker films, pointing to an efficient suppression of the electron–hole recombination (Figure 5b, inset).

**Figure 6:**
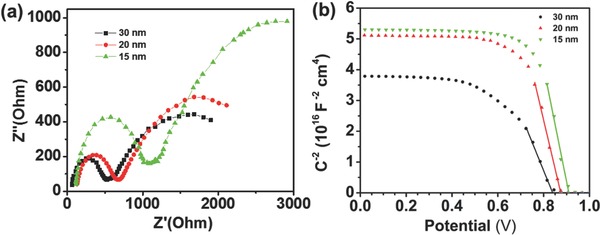
a) Nyquist and b) Mott–Schottky plots for the PVSK devices with 15, 20, and 30 nm thickness of the NiCo_2_O_4_ HTL.

To further understand the charge recombination processes during the hole collection process from perovskite to NiCo_2_O_4_ layer, we performed electro‐impedance spectroscopy (EIS) measurements under solar light and zero bias. **Figure** **6**a shows the characteristic Nyquist plots of the three corresponding PVSCs devices for 15, 20, and 30 nm sized NiCo_2_O_4_ films. The results showed a shape of two frequency responses for PVSCs, where the second semicircle (feature at low frequencies) is been attributed to the recombination resistance (Rrec).^74^, ^75^ As the NiCo_2_O_4_ HTL thickness is reduced the radius of the semicircle increases, this parameter implies a higher resistance in the charge recombination, in agreement with the findings from PL measurements. Figure 6b shows the M‐S plots of the devices when sweeping from higher to a lower voltage. The crossing of the curves at 1/*C* ^2^ = 0 is attributed to the flat band potential of the device, while the lower slope of the linear region is attributed to the charge accumulation at the interfaces, which impedes an efficient extraction of the charge carriers.^76^ The M‐S slope for the thicker film is lower implying that this layer cannot extract fast enough the charge carriers, inducing their accumulation at the interface. This behavior causes a higher hysteresis, which in turn increase electron–hole recombination (due to high spatial density) and leads to the drop of the flat band potential. On contrary, the thinner NiCo_2_O_4_ HTL seems to extract faster the charge carriers, increasing the flat band potential, and thus the *V*_oc_, as well as the FF of the corresponding device. The direct correlation of faster charge carriers extraction at thinner HTL layers with increased FF has been previously studied by Stolterfoht et al.^77^ We also notice that the configuration of 15 nm NiCo_2_O_4_ HTL is depleted faster than the other devices due to enhanced charge carrier collection, which can also confirm the increase at the FF of the corresponding PVSC. The increased *J*_sc_ for the 15 nm NiCo_2_O_4_ layer can be ascribed to the higher transparency of the thin film compared to thicker HTLs (shown above) resulting to enhanced photogeneration of electron–hole pairs as well as to the lower series resistance as shown in Table 1.

Importantly, improved reproducibility and performance reliability of the NiCo_2_O_4_‐HTL p‐i‐n PVSCs, compare to our previously reported solvothermal synthesized CuO‐HTL based PVSCs^22^ was demonstrated by applying combustion synthesized NiCo_2_O_4_ HTLs. Specifically, as shown in Figure S5 (Supporting Information) the combustion synthesized NiCo_2_O_4_‐HTL delivers a ≈14.5% average PCE (16 devices) with 15.5% best performing device, while CuO‐HTL based solar cells give a ≈12.5% average PCE (16 devices) with 15.3% best performing device.^22^

## 3 Conclusions

In conclusion, a low‐temperature combustion synthesis method, using for the first time a tartaric acid as a fuel, was successfully developed and applied for the fabrication of compact films of p‐type NiCo_2_O_4_ NPs. The size of the NPs was fully controlled due to the usage of tartaric acid leading to the formation of monodispersed NiCo_2_O_4_ NPs with a diameter of ≈4 nm. The combustion proceeds under low temperature (250 °C) and within a short reaction time (1 h), produce high‐quality, homogeneous NiCo_2_O_4_ NPs films with high electrical conductivity (≈4 S cm^−1^) and very low roughness (0.56 nm) functional layers were fabricated. The detailed physicochemical characterization of the NiCo_2_O_4_ NPs using XRD, EDS, and electron microscopy measurements confirm the high purity, crystallinity, and small grain composition of the NiCo_2_O_4_. Furthermore, the proposed synthetic approach allowed the production of compact films using blade coating, which is a large‐scale compatible technique appropriate for the development of printed electronic devices. The impact of NiCo_2_O_4_ HTL thicknesses on PVSCs characteristics was also investigated. The optimum thickness is found to be 15 nm showing enhanced charge carrier collection and negligible *J*–*V* hysterics, compared to thicker films, delivering reliable p‐i‐n PVSCs with a PCE of 15.5%. We believe that the proposed combustion synthesis method using a tartaric acid as a fuel can provide a route to produce highly reproducible metal oxides suitable for use in a range of advanced materials applications.

## 4 Experimental Section

*Materials*: Prepatterned glass‐ITO substrates (sheet resistance 4Ω sq^−1^) were purchased from Psiotec Ltd., Pb(CH_3_CO_2_)_2_.3H_2_O from Alfa Aesar, methylammonium iodide (MAI) and methylamonium bromide (MABr) from Dyenamo Ltd., PC[70]BM from Solenne BV. All the other chemicals used in this study were purchased from Sigma‐Aldrich.

*Synthesis of NiCo_2_O_4_ NPs Films*: For the combustion synthesis of NiCo_2_O_4_ NPs, 0.5 mmol Ni(NO_3_)_2_.6H_2_O, 1 mmol Co(NO_3_)_2_.6H_2_O, and tartaric acid were mixed in the 15 mL 2‐methoxy ethanol solution. After 150 uL HNO_3_ (69 wt% HNO_3_) were added slowly into the mixture, and the solution stirred up to almost complete homogeneity. The whole solution was allowed under stirring for 30 min at 60 °C. The ratio of the total metal nitrates and tartaric acid was 1. Thereafter, the violet colored solution was used for the combustion synthesis of the NiCo_2_O_4_ NPs on the various substrates. Doctor blade technique was applied for the fabrication of the precursor films on the various substrates. The resulting light violet colored films were dried at 100 °C for 30 min, and used as a precursor for the combustion synthesis of NiCo_2_O_4_ NPs. Subsequently, the obtained films were heated at different temperatures (200, 250, and 300 °C) in ambient atmosphere for 1 h in a preheated oven to complete the combustion process and then left to cool down at room temperature. For UV–vis absorption measurements, the films were fabricated on quartz substrates, while for the transmittance measurements 30, 20, and 15 nm thick films were fabricated on glass/ITO substrates applying 250 °C heating temperature, respectively.

*Device Fabrication*: The inverted solar cells under study was ITO/NiCo_2_O_4_‐NPs/CH_3_NH_3_PbI_3_/PC[70]BM/Al. ITO substrates were sonicated in acetone and subsequently in isopropanol for 10 min and heated at 100 °C on a hot plate 10 min before use. The perovskite solution was prepared 30 min prior spin coating by mixing Pb(CH_3_CO_2_)_2_.3H_2_O:methylamonium iodide (1:3) at 36 wt% in dimethylformamide (DMF) with the addition of 1.5% mole of MABr.^78^, ^79^, ^80^ The precursor was filtered with 0.1 µm polytetrafluoroethylene (PTFE) filters. The perovskite precursor solution was deposited on the HTLs by static spin coating at 4000 rpm for 60 s and annealed for 5 min at 85 °C, resulting in a film with a thickness of ≈230 nm. The PC[70]BM solution, 20 mg mL^−1^ in chlorobenzene, was dynamically spin coated on the perovskite layer at 1000 rpm for 30 s. Finally, 100 nm Al layers were thermally evaporated through a shadow mask to finalize the devices giving an active area of 0.9 mm^2^. Encapsulation was applied directly after evaporation in the glove box using a glass coverslip and an Ossila E131 encapsulation epoxy resin activated by 365 nm UV irradiation.

*Characterization*: TGA and differential thermal analysis (DTA) were performed on a Shimadzu Simultaneous DTA‐TG system (DTG‐60H). Thermal analysis was conducted from 40 to 600 °C in air atmosphere (200 mL min^−1^ flow rate) with a heating rate of 10 °C min^−1^. XRD patterns were collected on a PANanalytical X'pert Pro MPD powder diffractometer (40 kV, 45 mA) using Cu Kα radiation (λ = 1.5418 Å). TEM images and electron diffraction patterns were recorded on a JEOL JEM‐2100 microscope with an acceleration voltage of 200 kV. The samples were first gently ground, suspended in ethanol and then picked up on a carbon‐coated Cu grid. Quantitative microprobe analyses were performed on a JEOL JSM‐6390LV scanning electron microscope equipped with an Oxford INCA PentaFET‐x3 EDS detector. Data acquisition was performed with an accelerating voltage of 20 kV and 60 s accumulation time. Transmittance and absorption measurements were performed with a Schimadzu UV‐2700 UV–vis spectrophotometer. The thickness of the films was measured with a Veeco Dektak 150 profilometer. The current density–voltage (*J*/*V*) characteristics were characterized with a Botest LIV Functionality Test System. Both forward and reverse scans were measured with 10 mV voltage steps and 40 ms of delay time. For illumination, a calibrated Newport Solar simulator equipped with a Xe lamp was used, providing an AM1.5G spectrum at 100 mW cm^−2^ as measured by a certified oriel 91150 V calibration cell. A shadow mask was attached to each device prior to measurements to accurately define the corresponding device area. EQE measurements were performed by Newport System, Model 70356_70316NS. AFM images were obtained using a Nanosurf easy scan 2 controller under the tapping mode. Electrical conductivity measurements were performed using a four‐point microposition probe, Jandel MODEL RM3000. EIS and M‐S measurements were performed using a Metrohm Autolab PGSTAT 302N, where for the EIS a red light‐emitting diode (at 625 nm) was used as the light source calibrated to 100 mW cm^−2^. For EIS a small AC perturbation of 20 mV was applied to the devices, and the different current output was measured throughout a frequency range of 1 MHz to 1 Hz. The steady state DC bias was kept at 0 V throughout the EIS experiments.

## Conflict of Interest

The authors declare no conflict of interest.

## Supporting information

Supplementary
