# Supplementary material for: Low‐Temperature Combustion Synthesis of a Spinel NiCo2O4 Hole Transport Layer for Perovskite Photovoltaics
Source: Adv Sci (Weinh). 2018 Mar 3;5(5):1701029. doi: 10.1002/advs.201701029 (PMC5979620; doi:10.1002/advs.201701029)
Supplement: Supplementary file 1 — Supplementary [file ADVS-5-1701029-s001.pdf]

Copyright WILEY-VCH Verlag GmbH & Co. KGaA, 69469 Weinheim, Germany, 2018.

ADVANCED SCIENCE
Open Access

Supporting Information

for *Adv. Sci.,* DOI: 10.1002/advs.201701029

Low-Temperature Combustion Synthesis of a Spinel $NiCo_2O_4$ Hole Transport Layer for Perovskite Photovoltaics

*Ioannis T. Papadas, Apostolos Ioakeimidis, Gerasimos S. Armatas, and Stelios A. Choulis**

Supporting information

Low Temperature Combustion Synthesis of a Spinel $NiCo_2O_4$ Hole Transport Layer for Perovskite Photovoltaics

Ioannis T. Papadas,[a] *Apostolos Ioakeimidis,*[a] *Gerasimos S. Armatas*[b] *and Stelios A. Choulis*[a]*

[a] Molecular Electronics and Photonics Research Unit, Department of Mechanical Engineering and Materials Science and Engineering, Cyprus University of Technology, Limassol, Cyprus.

[b] Department of Materials Science and Technology, University of Crete, Heraklion 71003, Greece.

*Corresponding Author: Prof. Stelios A. Choulis

E-mail: stelios.choulis@cut.ac.cy

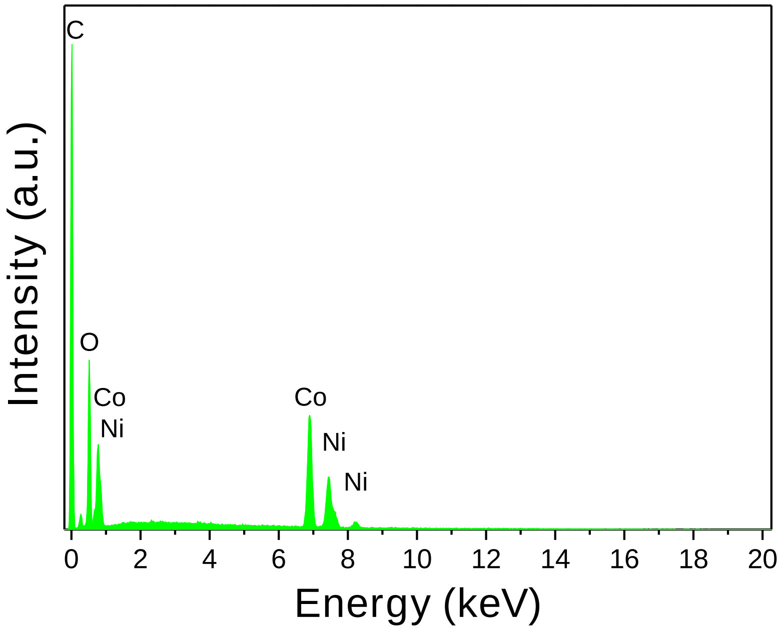

Figure S1. Typical EDS spectrum for $NiCo_2O_4$ nanoparticles. The EDS analysis indicates an average atomic proportion of Ni:Co ~ 1:2.

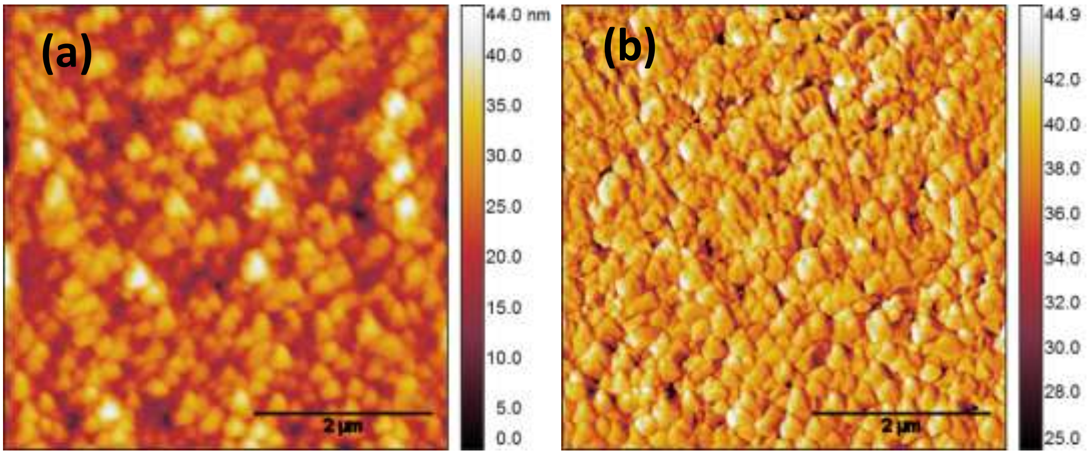

Figure S2. (a) Surface topography and **(b)** the corresponding phase image (5x5 µm) of 230 nm Perovskite film obtained by AFM. The film exhibit a roughness (root mean square) of 5.4 nm.

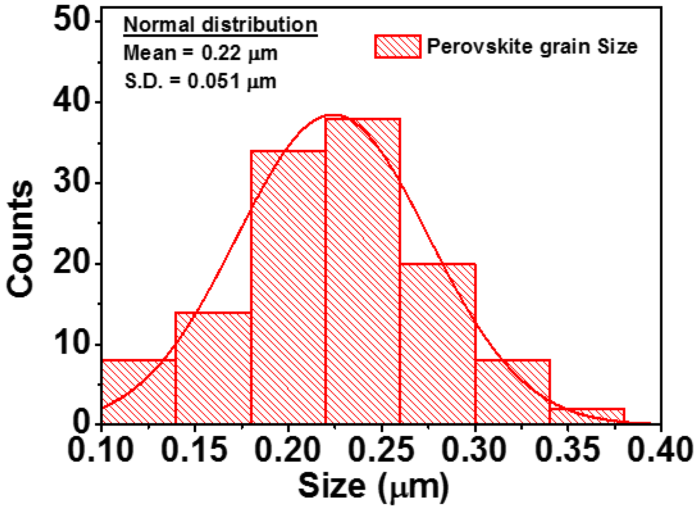

Figure S3. Size distribution of perovskite grains extracted from the AFM topography images. The mean size of the grains is 0.22 µm with a standard deviation of 0.051 µm.

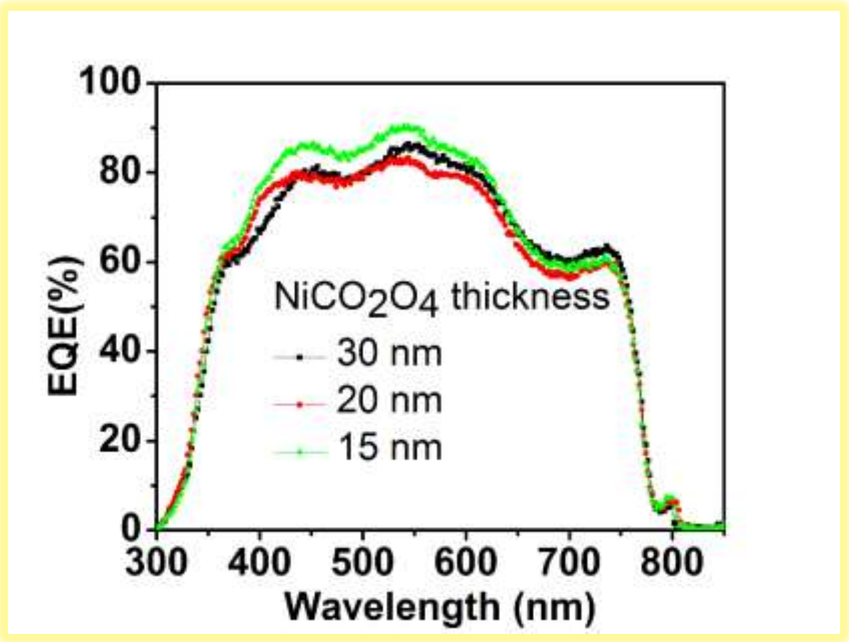

Figure S4. External Quantum Efficiency (EQE) of ITO/$NiCo_2O_4$-NPs/$CH_3NH_3PbI_3$ devices fabricated with $NiCo_2O_4$ with different thickness (15 nm - green solid line, 20 nm - red line and 30 nm – black line).

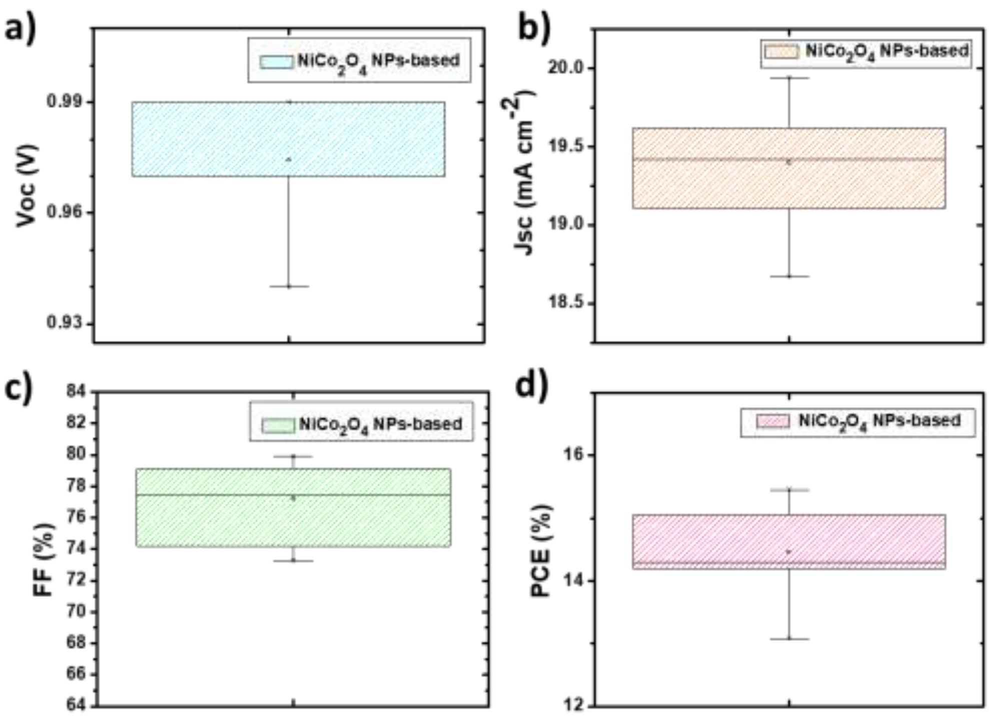

Figure S5. Average photovoltaic parameters represented in box plots out of 16 devices of each series of p-i-n perovskite solar cells under study. $NiCo_2O_4$ NPs-based

devices with box plots, a) open circuit voltage (Voc), b) current density (Jsc), c) fill factor (FF) and d) power conversion efficiency (PCE).
